# Effectiveness of Interventions to Promote Physical, Psychological, and Socioeconomic Well-being Outcomes of Parents of Children With Neurodevelopmental Disabilities: Protocol for a Systematic Review

**DOI:** 10.2196/38686

**Published:** 2022-07-28

**Authors:** Monika Novak-Pavlic, Peter Rosenbaum, Luciana Gazzi Macedo, Briano Di Rezze, Joshua Yong, Atefeh Noori, Debra Hughes

**Affiliations:** 1 School of Rehabilitation Science McMaster University Hamilton, ON Canada; 2 CanChild Centre for Childhood Disability Research McMaster University Hamilton, ON Canada; 3 Department of Pediatrics Faculty of Health Sciences McMaster University Hamilton, ON Canada; 4 Department of Occupational Therapy Sengkang General Hospital Singapore Singapore; 5 Toronto Rehabilitation Institute Toronto, ON Canada

**Keywords:** childhood disability, developmental disability, family, health, parent intervention, pediatric, children, parenting, rehabilitation, child development, health intervention, peer support, socioeconomic well-being, parent support, meta-analysis, quality of life

## Abstract

**Background:**

It is well recognized that parents of children with neurodevelopmental disabilities can experience a considerable burden of care associated with their child’s disability, which can potentially impact their functioning and quality of life. Historically, the intervention efforts in pediatric rehabilitation have focused primarily on the child’s development and well-being and much less on parental and family well-being. The impact that a child’s diagnosis might have on parents remains unclear, and it is unknown how we can best support parents on their journey of childhood disability. It is, therefore, important to synthesize the published evidence on interventions for parents of children with neurodevelopmental disabilities so that clinicians can be better informed about the ways in which families they work with can be supported.

**Objective:**

This manuscript presents the protocol for a systematic review of the effectiveness of interventions aiming to improve the physical, psychological, or socioeconomic well-being of parents of children with neurodevelopmental disabilities when compared to usual care or no care.

**Methods:**

We will systematically search 4 databases (MEDLINE, Embase, PsycINFO, and CINAHL) from the year 2000 until the search date, for randomized controlled trials that evaluated the effectiveness of interventions to improve parental physical, psychological, or socioeconomic well-being. Two authors will independently screen the titles and abstracts, which will then be followed by full-text screening. After the eligibility assessment, two reviewers will independently extract data and conduct a risk of bias assessment using the Cochrane risk-of-bias tool. We will assess the quality of evidence using the Grading of Recommendations, Assessment, Development and Evaluation approach. If the data allow, we will perform a pairwise meta-analysis or network meta-analysis. We plan to evaluate the coherence of the network with a global test by using the node-splitting method.

**Results:**

As of May 30, 2022, there have been two searches of data initiated: in September 2020 for articles published since 2000 and an updated search in January 2022 for articles published since 2020. We have screened all the titles and abstracts and performed eligibility assessment. However, the final number of references is still not available due to the additional information needed for some of the potentially eligible studies. The results from this systematic review will be published in an indexed journal within a year after this protocol is published.

**Conclusions:**

This study is expected to identify a variety of programs to address the well-being needs of parents of children with neurodevelopmental disabilities and provide directions on how parents can best be supported within health care. Such interventions might help professionals and stakeholders in creating service delivery models that can enhance parental well-being and minimize the risks to their physical, psychological, and socioeconomic functioning.

**Trial Registration:**

PROSPERO CRD42021230706; https://www.crd.york.ac.uk/prospero/display_record.php?RecordID=230706

**International Registered Report Identifier (IRRID):**

DERR1-10.2196/38686

## Introduction

### Background

Becoming a parent of a child with a disability can be a life-altering experience, often accompanied by rearranging family life and functioning [1]. How parents adapt to this unexpected role and its associated caregiving demands is highly individual and influenced by many factors [2]. Caring for a child who has a disability often has an impact on caregiver health, functioning, and well-being. Despite the positive side of being a parent of a child with a disability [1,3-5], research consistently shows that these parents are more likely to experience challenges in their physical, psychological, financial, and social functioning [6-12]. Some of those challenges, such as the increased levels of stress and higher risk for depression (relative risk 1.75, 95% CI 1.55-1.97) and anxiety (1.40, 95% CI 1.18-1.67), have been extensively explored and are consistently found to be directly related to the increased burden of care for their child(ren) [7,8].

Furthermore, research shows that parenting a child with a disability does not only bring challenges and risks concerning psychological functioning and mental health; there is evidence that having a child with a disability might also affect parents’ physical health and longevity. Research shows that parent caregivers of children who depend on medical technology are at risk of sleep deprivation, sleep disturbances, and excessive daytime sleepiness, which can all have serious consequences on health, daytime functioning, and quality of life [13,14]. Similar results were also found with parents of children with autism [7]. In addition, intensive physical demands of caregiving for children with physical disabilities can also lead to increased back pain [15-17]. A population-based cohort study by Cohen et al [9,10] showed that mothers of children with a congenital anomaly have increased cardiovascular risk and even increased risk of premature death when compared to other mothers.

In addition, parents of children with impairments have been found to experience more social marginalization, financial difficulties [18], under- or unemployment [7], and other disadvantages. We believe that parents’ life circumstances can have serious impact on their overall well-being and prosperity. It is, therefore, important to identify which interventions effectively address parental needs and well-being, so that we can learn what, how, where, and for whom interventions have been explored.

Until recently, there has been no published model or framework that could guide professionals in considering parental well-being in the context of pediatric rehabilitation service delivery and research exploration. A recent scoping review by King et al [19] provided a framework that encompasses types of services that promote parent and family wellness. This study has been a significant contribution to the field by determining the types of services that parents and families need. It can be used as a cornerstone for mapping out and classifying existing services, as well as for guiding research exploration and implementation in day-to-day clinical practice. By building on this foundation, we want to expand on one of its domains, *addressing parent-specific needs*, and explore what interventions have focused specifically on parental health outcomes, functioning, needs, and well-being.

This paper presents the protocol for a systematic review of randomized controlled trials (RCTs) that aimed to evaluate the effectiveness of interventions addressing parental needs and well-being with a focus on physical, psychological, and socioeconomic outcomes. At the end of our proposed study, we will (1) report on the RCTs within the literature that explored the effectiveness of interventions aiming to support parents, and (2) inform about ways in which clinicians can better support the well-being and needs of parents of children with disabilities in everyday practice.

### Objective

The objective of this study is to examine the effectiveness of interventions addressing parent-specific needs and the well-being of parents of children with disabilities. The following research question will be addressed:

What is the effectiveness of interventions focusing on parent-specific needs for parents of children with neurodevelopmental disabilities on physical, psychological, and socioeconomic well-being outcomes when compared to no care or usual care?

## Methods

### Overview

This protocol was written according to the PRISMA-P (Preferred Reporting Items for Systematic Review and Meta-Analysis Protocols) 2015 checklist [20] and the PRISMA for Network Meta-Analyses extension statement [21]. In accordance with the guidelines, this protocol was registered with PROSPERO (International Prospective Register of Systematic Reviews; 230706).

### Ethical Considerations

Since this study will not involve contact with participants, we did not seek ethics approval.

### Eligibility Criteria

#### Study Design

We will include RCTs, including feasibility RCTs, that investigated interventions that directly address physical, psychological, or socioeconomic well-being and needs of parents of children with disabilities. In the absence of RCTs (n<3), we will also include quasi-randomized studies. Only peer-reviewed studies will be eligible for inclusion (full-length reports only, not abstracts or conference proceedings).

#### Intervention

We will include studies that explored programs or interventions that were designed to address parent-specific physical, psychological, and/or socioeconomic needs, health, and well-being. We will exclude studies that were primarily child-focused but had a parent training, education, or assessment component. We will also exclude studies examining parent-mediated interventions for managing their child’s symptoms or disability. Articles that did not describe the components of their intervention will be excluded. Additionally, studies that explored pharmacological or diet interventions will not qualify for this review. Studies will be eligible if the comparison interventions include no intervention, usual care, or wait-list control. Studies with multiple intervention arms (>2) will be considered only if there was a usual care, nonexposed, or wait-list control group.

#### Population

The population of interest in this study is parents of children (0-18 years) with lifelong neurodevelopmental disabilities (eg, cerebral palsy, Down syndrome, autism spectrum disorder, spina bifida, intellectual disabilities, multiple disabilities, genetic syndromes). This does not include parents of children who have mental illness (eg, depression, anxiety), eating disorders, behavioral problems (eg, oppositional defiant disorder, emotional disorders), or chronic diseases (eg, epilepsy, cancer, asthma) only. We will use the Institute of Medicine (US) Committee on Nervous System Disorders in Developing Countries [22] definition of developmental disabilities to guide our search strategy and define our target study population. According to the Institute of Medicine (US) Committee on Nervous System Disorders in Developing Countries, (neuro)developmental disabilities “include limitations in function resulting from disorders of the developing nervous system” [22], such as (1) cognitive (eg, intellectual disability, learning disability), (2) motor (eg, cerebral palsy, spina bifida), (3) vision (eg, blindness, visual impairment), (4) hearing and speech (eg, deafness, hearing impairment), and (5) behavior (eg, attention-deficit/hyperactivity disorder, autism spectrum disorder) disabilities [22]. We will include reports that focus primarily on parents and where parents of children with disabilities comprised 90% of the sample. In studies where there are multiple populations of interest (eg, parents, clinicians, teachers, grandparents), we will include them if at least 90% of parents were raising children with disabilities and will focus only on parent-specific data, analysis, and results. At least 90% of children of the parents included in the studies need to be children with lifelong disabilities who are aged ≤18 years.

#### Outcomes

We will consider parent-specific outcomes that fall under one of the following categories: physical well-being (eg, pain, fatigue), psychological functioning (eg, mental health, anxiety, depression, stress, self-efficacy, self-esteem, empowerment), and socioeconomic outcomes (eg, financial status, employment, friendships, sense of belonging, social support). Outcomes must be assessed by a validated tool or standardized measurement to be eligible for inclusion. We will extract data for individual outcomes, as reported in the original report.

### Information Sources

We will search four electronic bibliographic databases: MEDLINE (1946-), PsycINFO (1987-), Embase (1984-), and CINAHL. We will also hand screen the reference lists of identified reviews and assess them for eligibility. Since the field of childhood disability has significantly changed in the last two decades due to advancements in health care thinking and societal changes, we will include only studies published since 2000. We will not apply any language or location criteria. Identified master’s or doctoral dissertations that satisfy the eligibility criteria will also be considered for inclusion.

### Search Strategy

Literature searches will include both subject headings and keywords with regard to the population, intervention, outcomes, and study design. The search strategy will be developed in collaboration with an experienced university librarian. A draft of the MEDLINE search strategy can be found in Table 1. The search might be rerun toward the end of the review.

**Table 1:** MEDLINE search.

	Search terms
P (population): parents of children with developmental disabilities	Parents/, Parent*.mp., Mothers/, mother*.mp., Fathers/, father*.mp. (infant* or child* or teenage* or adolescen*).mp., Infant/ or child/ or adolescent/ (disab* or disorder*).mp., Disabled Children/ Child Development Disorders/, Pervasive/ or development* disorder*.mp. or Developmental Disabilities/, Neurodevelopmental Disorders/ or neurodevelopment* dis*.mp., Cerebral Palsy/ or cerebral palsy.mp., Spinal Dysraphism/ or spina bifida.mp. or spinal dysraphism.mp., Muscular Dystrophies/ or Muscular Dystrophy, Duchenne/ or muscular dystroph*.mp., Autistic Disorder/ or autis*.mp. or Autism Spectrum Disorder/, Asperger Syndrome/ or asperger syndrome*.mp., Attention Deficit Disorder with Hyperactivity/ or ADHD.mp. or attention deficit disorder*.mp., hyperactiv*.mp., Down Syndrome/ or Down syndrome*.mp., deaf*.mp. or Deafness/ or Deaf- Blind Disorders/ or blind*.mp. or Blindness/, Vision Disorders/ or vision disorder*.mp. or Visually Impaired Persons/ or visual impairment*.mp. or or deaf*blind*.mp., Intellectual Disability/ or intellectual dis*.mp.
I (intervention): any	(intervention* or service* or program*).mp., (treatment* or therap*).mp., Mentoring/ or mentor*.mp., Counseling/ or counsel*.mp., support group*.mp., (peer-to-peer or peer to peer).mp.
O (outcomes): physical, psychological, and socioeconomic outcomes	pain*.mp. or Pain/, discomfort.mp., Fatigue/ or fatigue.mp., (burnout or burn out or burn-out).mp. or Burnout, Psychological/, Anxi *.mp. or Anxiety/ or Anxiety Disorders/, Depression/ or depress*.mp., Stress, Psychological/ or stress*.mp., confiden*.mp., Self Efficacy/ or self*efficacy.mp., empower*.mp. Motivation/ or motivat*.mp., Mental Health/ or mental health.mp., worr*.mp., (wellbeing or well being or well- being).mp., Sick Leave/ or sick leave.mp., quality of life.mp. or “Quality of Life”/, productiv*.mp., Income/ or income.mp. or Poverty/, relationship*.mp., expense*.mp., Employment/ or employ*.mp., work.mp. or Work/, Job Satisfaction/ or Job Application/ or job*.mp., Social Support/ or social support.mp.
Study design: randomized controlled trials, quasi-randomized trials, mixed method trials	(RCT* or randomized controlled trial*).mp. or (controlled trial* or controlled stud*).mp, (quasi-randomized or quasi-experimental*).mp., (quasi- randomized or quasi- experimental*)-.mp., (mixed-method* or mixed method*).mp.

### Study Records

References yielded from the databases will be uploaded to Covidence Systematic Review Software [23]. We will use this web-based software program for deduplication and the first two levels of assessments: (1) title and abstract screening, and (2) full-text screening and eligibility assessment. Deduplication will be done automatically using Covidence and manually through the inspection of the reference list of identified reviews. The first author will manually upload the full-text reports after the first-level screening is completed. Data from the included studies will be extracted and stored in a Microsoft Excel spreadsheet [24]. Possible quantitative analysis will be done using the statistical software Stata (release 15.1; StataCorp LLC) [25] or R (R Foundation for Statistical Computing) [26].

### Study Selection

Prior to starting to screen, a calibration exercise will be performed on at least 150 reference titles and abstracts to ensure consistency among the reviewers. Two reviewers (MNP and JY) will then independently screen titles and abstracts. Potentially eligible articles will move to the full-text screening phase. The two authors will then independently perform full-text screening for eligibility. Discrepancies in decisions between the reviewers will be resolved through discussion. If an agreement cannot be achieved, a third reviewer will be included. Additional information from study authors will be sought if critical information about the study is missing and the reviewers are unclear about its eligibility.

In cases where articles were published in languages that the two authors do not speak, we will consult with a native or bilingual colleague (researcher or graduate trainee) to determine its eligibility by looking at the full text. If a non-English full report is eligible, we will arrange an external translation service to create an English-language version before any data are extracted. Throughout the entire data extraction and interpretation process, we will continuously consult with colleagues fluent in that language to ensure accuracy.

Data extraction will be carried out in duplicate by two authors using a detailed data extraction manual. Any potential disagreements will be resolved through discussion. If further information about the study is required, we will contact the study authors directly (3 email attempts). If we identify multiple reports of a single study, we will treat them as one study.

### Data Items

The following data will be extracted for the included articles:

- General information about the study (authors, year of publication, location, journal or other site where the record was published, study aim/research question)
- Study details (study design, unit of allocation, randomization/sampling strategy, number of groups)
- Information about the participants (population description, sample size, demographics, types of childhood disabilities, children’s age, control)
- Information about the intervention—modified Template for Intervention Description and Replication Checklist [27] (name and type of intervention, intervention description, materials/procedures, providers, setting in which the intervention occurred, duration, frequency, dose/intensity, format and mode of delivery, tailoring, modifications, fidelity, follow-up)
- Information about the parent-specific outcomes assessed (outcome name and definition, measures used, data collection time points, type of outcome [dichotomous, ordinal, continuous], baseline and first follow-up scores for continuous outcomes [mean, standard deviation, sample size] and number of events and sample size for dichotomous outcomes)
- Other information (key conclusions of study authors, adverse events, correspondence required)

### Outcomes and Prioritization

Parents of children with disabilities can face challenges in multiple areas of life and functioning. In this study, we are interested to learn about the effects that interventions might have on three domains of well-being: *physical*, *psychological*, and/or *socioeconomic* well-being. We will extract data for outcomes that fit under one or more of these three categories.

For the purposes of this review, *physical outcomes* will be explicitly related to parents’ physical health (eg, pain, muscle strength, cancer). An outcome will be considered *psychological* when it represents personal values, perceptions, judgments, feelings, or evaluations (eg, self-esteem, confidence, mental health, coping, self-efficacy). *Socioeconomic well-being outcomes* include various domains, such as social health and functioning and financial stability (eg, social support, relationships, employment). The judgment for inclusion of each outcome, and where it fits in the physical-psychological-​socioeconomic map, will be done separately by two reviewers during the extraction phase. Potential discrepancies will be discussed.

### Risk of Bias and Quality Assessment

The risk of bias will be assessed using the revised Cochrane risk-of-bias tool for randomized trials [28]. Each study will be evaluated with respect to sequence generation, allocation concealment, blinding, incomplete outcome data (≥20% missing data will be considered to be at high risk of bias), selective reporting, and other biases [28]. According to available information in the report, a judgment as to whether there is “high risk,” “low risk,” or “unclear risk” of bias will be made independently by two review authors. All potential disagreements in judgments will be discussed. The outcomes of the risk of bias assessment will be visually presented using RevMan [29] or Robvis [30].

The quality assessment of the outcomes of interest from the included studies will be done using the Grading of Recommendations Assessment, Development and Evaluation (GRADE) approach [31]. We will first rate the importance of relevant outcomes in the literature as either *critical*, *important but not critical*, or *of limited importance* [32]. We will use the GRADE approach for rating the quality of evidence for each outcome or network estimate on an outcome-by-outcome basis. Network estimates quality will be rated based on the direct or indirect evidence that has contributed most to the network and will be rated as high, moderate, low, or very low quality based on the limitations in risk of bias, imprecision, inconsistency, indirectness, and publication bias. We will present the results of our quality assessment in a Summary of Findings table.

### Data Synthesis

Data will be pooled if studies are homogeneous with respect to the intervention and outcomes measured. For each direct comparison, we will calculate standardized mean differences for the continuous outcomes or weighted mean differences if the outcomes are the same, and odds ratio for dichotomous outcomes with the associated 95% confidence intervals. Statistical heterogeneity will be assessed by visual inspection of forest plots and the I² statistic. I² of 0%-40% will be considered as “might not be important,” 30%-60% as “moderate,” 50%-90% as “substantial,” and 75%-100% as “considerable” based on the Cochrane Handbook [33].

We will use the before and first follow-up measurements in cases of more than one assessment. We will perform a pairwise meta-analysis of the available direct comparisons using the DerSimonian–Laird random effects model for all outcomes. We will perform network meta-analysis to synthesize the available evidence from the entire network of trials by integrating direct and indirect estimates for each comparison into a single summary treatment effect. We will apply a frequentist random effects model using the methodology of multivariate meta-analysis to assess the comparative effectiveness of eligible interventions [34] in Stata [25].

We will perform a network meta-analysis to compare the effectiveness of identified interventions if the assumption of transitivity is met, meaning that “there are no systematic differences between the available comparisons other than the treatments being compared” [35]. The transitivity assumption will be assessed by investigating effect modifiers. If transitivity is not demonstrated, we will consider building separate networks for analysis. We will create network graphs to visualize the geometry of the networks and evaluate the networks’ statistical incoherence with global design-by-treatment and local approaches using the node-splitting method. We will assume a common heterogeneity estimate across the network.

If conventional meta-analysis and network meta-analysis cannot be done for any methodological or data-related reason(s), the results will be presented qualitatively. We will use summary tables and figures to present our results.

## Results

The first search of the literature of this systematic review commenced in September 2020 (from 2000). Due to the longevity of the process and limited resources, we ran an updated search on January 23, 2022 (from 2020). Data extraction for the first round of screening was initiated in June 2021. After the updated search was run and reference screening was completed in March 2022, we also started the data extraction for the remaining articles, a process that is currently underway. As of May 30, 2022, we have extracted the majority of data that were available in reports but are still in contact with the original authors of some of the studies for additional information. For example, we reached out to some authors to ask about the availability of a data subset or subgroup analysis, as well as some further details about the study execution that will confirm the studies’ eligibility and direct our analytic approach. We plan to conduct the analysis in June and July 2022 and publish the results of this study within one year of publishing this protocol.

## Discussion

### Overview

This systematic review will provide a better understanding of the effectiveness of available programs that can be used to support the physical, psychological, and socioeconomic well-being of parents of children with neurodevelopmental disabilities. Based on the findings, we will determine the strength and quality of evidence for making recommendations for the implementation of evidence-based programs that support parents. Directions for future research will be discussed.

Research indicates that parents of children with neurodevelopmental disabilities face common challenges in their caregiving role across different types of childhood disability [36,37]. On the other hand, the realities of two families who both have, for example, a child on the autism spectrum might be hugely different. An approach that recognizes the commonalities of experiences of families of children with different types of disabilities, as well as the variations within the same disability category, is called *a noncategorical approach* [38]—an approach we have taken in this study. Despite the differences between families, evidence supports that parents value opportunities to connect with parents of children with different diagnoses and can greatly benefit from such opportunities [39]. Due to the expected heterogeneity of the population and interventions reported in eligible studies in this review, we anticipate that the statistical analysis will require multiple team discussions and careful consideration of all the factors (eg, study setting, population characteristics, type of intervention). Because of the breadth of our research question and lack of homogeneity of studies, we might be limited in our ability to conduct a robust statistical analysis and make strong clinical recommendations based on statistical findings. However, this study will provide a wide overview of the work that has been done to support parent-specific needs, with an aim of addressing their health and well-being. We also expect that this systematic review will identify gaps in the literature and provide direction for creating family-centered, needs-based, accessible solutions for supporting parent-caregivers.

### Amendments

All the potential changes to this protocol will be published as amendments on our PROSPERO protocol webpage. We will share the date of each amendment, the changes we make, and the rationale for them.

## Abbreviations

GRADE: Grading of Recommendations, Assessment, Development and Evaluation
PRISMA-P: Preferred Reporting Items for Systematic Review and Meta-Analysis Protocols
PROSPERO: International Prospective Register of Systematic Reviews
RCT: randomized controlled trial
